# Whole-Genome Sequencing Reveals Distinct Mutational Patterns in Closely Related Laboratory and Naturally Propagated *Francisella tularensis* Strains

**DOI:** 10.1371/journal.pone.0011556

**Published:** 2010-07-19

**Authors:** Andreas Sjödin, Kerstin Svensson, Marie Lindgren, Mats Forsman, Pär Larsson

**Affiliations:** 1 Division of CBRN Security and Defence, FOI - Swedish Defence Research Agency, Umeå, Sweden; 2 Department of Clinical Microbiology and Clinical Bacteriology, Umeå University, Umeå, Sweden; 3 Laboratory for Molecular Infection Medicine Sweden (MIMS), Umeå University, Umeå, Sweden; University of Hyderabad, India

## Abstract

The *F. tularensis* type A strain FSC198 from Slovakia and a second strain FSC043, which has attenuated virulence, are both considered to be derivatives of the North American *F. tularensis* type A strain SCHU S4. These strains have been propagated under different conditions: the FSC198 has undergone natural propagation in the environment, while the strain FSC043 has been cultivated on artificial media in laboratories. Here, we have compared the genome sequences of FSC198, FSC043, and SCHU S4 to explore the possibility that the contrasting propagation conditions may have resulted in different mutational patterns. We found four insertion/deletion events (INDELs) in the strain FSC043, as compared to the SCHU S4, while no single nucleotide polymorphisms (SNPs) or variable number of tandem repeats (VNTRs) were identified. This result contrasts with previously reported findings for the strain FSC198, where eight SNPs and three VNTR differences, but no INDELs exist as compared to the SCHU S4 strain. The mutations detected in the laboratory and naturally propagated type A strains, respectively, demonstrate distinct patterns supporting that analysis of mutational spectra might be a useful tool to reveal differences in past growth conditions. Such information may be useful to identify leads in a microbial forensic investigation.

## Introduction

Following the anthrax attacks of 2001, microbial forensics has emerged as a new scientific discipline dedicated to the investigation of biocrime and bioterrorism to link pathogen, crime, and perpetrator [1]. In molecular methods being developed to this end, selectively neutral genetic mutations, such as synonymous single nucleotide polymorphisms (SNPs) and extragenic tandem repeats [2] present advantages over non-neutral mutations to establish relationships between strains. Non-neutral characters are more prone to homoplasy (i.e. sharing of marker states for other reasons than ancestry) and less likely to accumulate at a constant rate, properties that may distort phylogenetic analyses. However, non-neutral mutations may also provide a different but potentially important aspect for microbial forensics. Since such mutations may reflect the selective forces experienced by a bacterium, they may also provide information on past propagation conditions.

Here, we investigate this possibility by comparing mutational patterns detected in three strains designated SCHU S4 (FSC237), FSC198 (SE-219), and FSC043 of *Francisella tularensis* subspecies *tularensis* (type A1). Due to its high virulence, ease of dissemination, low infectious dose, and previous weaponisation, this pathogen has been classified by the Centers for Disease Control and Prevention among the top six ‘Category A’ biological threat agents [3]. Type A strains (in particular subgroup A1) demonstrate high virulence to humans [4] compared to the two other subspecies holarctica (type B) and mediasiatica, and are almost entirely restricted to North America [5]. To date, the only exception to the North American geographical confinement of *F. tularensis* type A is a handful of isolates recovered in Europe: in western Slovakia in 1986 [6], and in a bordering area in Austria in 1990 (Gurycova unpublished). A recent genomic sequencing effort demonstrated that one of the recovered Slovakian strains, FSC198, is virtually identical and has been derived from SCHU S4 [7]. Data from the study also provided plausible evidence supporting that the European isolates indeed represent valid natural isolates and not events of laboratory contamination.

We sequenced the strain FSC043, which is another derivative strain of the SCHU S4. In contrast to the assumed natural propagation of FSC198, the FSC043 has been cultivated repeatedly on artificial media in laboratories during which it likely lost its pathogenicity for mice [8]. Detection of different mutational patterns between these strains would therefore support the possibility to infer differences in culture conditions from mutational data.

## Results

### Correction of *Francisella tularensis* subsp. *tularensis* strain SCHU S4 genome sequence

The genome sequence of *F. tularensis* subsp. *tularensis* strain SCHU S4 AJ749949.1 [9] available in GenBank [10] contained sequence errors in the form of SNPs and incorrect variable numbers of tandem repeats (VNTR), as identified recently by Chaudhuri et al. [7]. We have confirmed these errors and a corrected version of the genome sequence of SCHU S4 has been deposited in GenBank under accession number AJ749949.2.

### Identified mutational patterns

**Figure 1:**
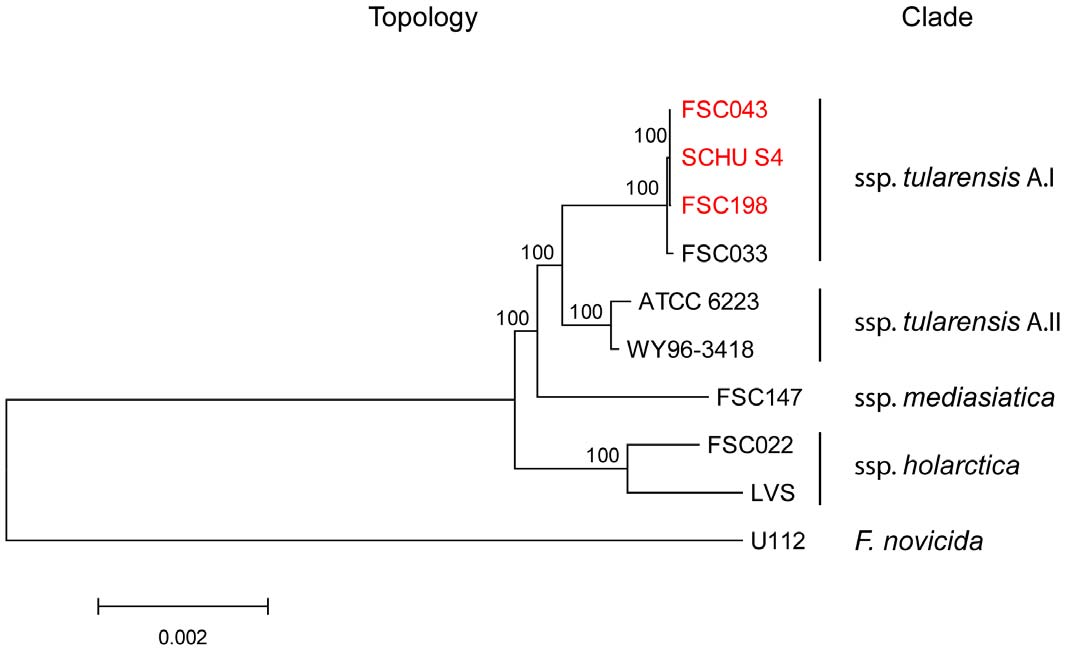
Relationships within the species *F. tularensis*. The evolutionary tree was inferred using the Neighbor-Joining method. Bootstrap support values (500 replicates) are shown next to branches. Scale bar indicates the number of base substitutions per site.

**Figure 2:**
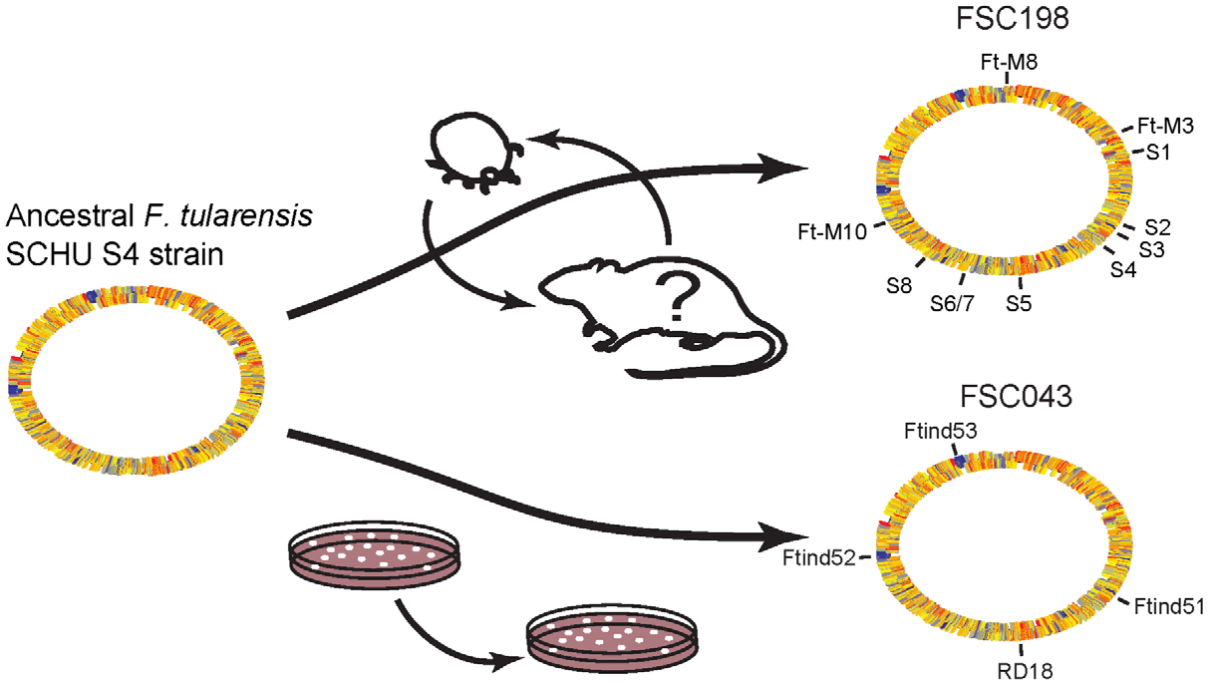
Overview of different paths of evolution. Strain FSC043 and strain FSC198 have been exposed to different environments since their split from the common ancestor strain SCHU S4. Strain FSC043 has experienced ‘artificial’ life cycles inside a laboratory while strain FSC198 has been exposed to a natural environment in Slovakia, which is reflected in their genomes by exhibiting different mutation patterns.

Direct mapping of sequence reads from FSC043 on the genome sequences of FSC198 and SCHU S4 showed an average coverage of 107× separated by highly repetitive regions. The phylogenetic positions and relationships of the analyzed strains are shown in Figure 1. Genome-wide sequence comparisons between strain FSC043 and strain SCHU S4 did not identify any SNPs between the two strains and they showed identical VNTR patterns, while three VNTRs differentiated them from FSC198. We found four insertion/deletion events differentiating FSC043 from SCHU S4 and FSC198 (Table 1). Three of them (Ftind51–53) were small deletions (2, 1, and 1 bp, respectively), while the fourth was a 1,480 bp deletion and corresponded to the previously identified RD18 [11]. Ftind51 affected a putative metal ion transporter protein (FTT0615), while Ftind52 and Ftind53 were located within the duplicated *Francisella* Pathogenicity Island (FPI). Additional sequencing confirmed Ftind52 and Ftind53 as single mutations in both copies of the *pdpC* gene (*pdpC1* and *pdpC2*). The eight previously identified SNPs (S1–S8) in FSC198 [7] were all non-synonymous mutations and affected genes for UDP-N-acetylglucosamine pyrophosphorylase (*glmU*), an outer membrane protein (FTT0602), an acid phosphatase (FTT0620), a soluble pyridine nucleotide transhydrogenase (*sthA*), a lipoprotein located between *lpn*A and *lpnB* (FTT0903), a cardiolipin synthetase (*ybhO*), and a D-methionine transport protein (FTT1124). A summary of mutations in the analyzed genomes (Table 2) is shown in Table 1. The proposed strain history and an overview of the mutations are depicted in Figure 2.

**Table 1:** Identified SNP, INDEL and VNTR differences between FSC198, FSC043 and SCHU S4 strains and their corresponding regions in three other genomes of *F. tularensis*.

Region^a^	Position^b^	Locus^b^	Gene^b^	FSC198	FSC043	SCHUS4	FSC033	WY96-3418	LVS
S1	390290	FTT0387	*glmU*	T	C	C	C	C	C
S2	621377	FTT0602c		T	C	C	C	C	C
S3	635510	FTT0620		A	C	C	C	C	C
S4	701627	FTT0684c	*sthA*	T	G	G	G	G	G
S5	911510	FTT0903		T	C	C	C	C	C
S6	1007563	FTT0997	*ybhO*	C	G	G	G	G	G
S7	1008148	FTT0997	*ybhO*	A	G	G	G	G	G
S8	1134416	FTT1124	*metN*	A	G	G	G	G	G
Ft-M3	8266	ISFtu1		14	21	21	14	2	13
Ft-M8	308634	FTT1124		5	4	4	2	2	2
Ft-M10	1083657	ISFtu1		11	18	18	6	1	2
Ftind51^d^	635249	FTT0615c			DEL^c^				
Ftind52^d^	1393671	FTT1354	*pdpC1*		DEL^c^				
Ftind53^d^	1787006	FTT1709	*pdpC2*		DEL^c^				
RD18	928574	FTT0918, FTT0919			DEL^c^				DEL^c^

a Regions S1–S8, Ft-M3, Ft-M8, Ft-M10 and RD18 have been published previously [5], [7], [11].

b Genes and positions are given according to SCHU S4 (AJ749949.2).

c DEL indicates deletion compared to other included strains.

d The Ftind-numbering continues the serial presented in [39]–[41].

**Table 2:** *F. tularensis* genome sequences used in this study.

Species	Subspecies	Strain	Alt. name	Origin	GenBank
*F. tularensis*	*tularensis* A1				
		FSC198	SE-219	Mite, Bratislava, Slovakia, 1988	AM286280.1
		FSC043		Attenuated SCHU S4 phenotypic variant	
		SCHU S4^a^	FSC237	SCHU phenotypic variant, 1951	AJ749949.2
		FSC033	SnMF	Squirrel, GA, USA, 1992	AAYE00000000.1
	*tularensis* A2				
		WY96-3418		Human ulcer, WY, USA, 1996	CP000608.1
	*holarctica*				
		LVS	ATCC 29684	Live vaccine strain, NDBR lot 11	AM233362.1

a SCHU S4 was derived from SCHU in 1951 by Henry T. Eigelsbach [42]. SCHU was originally isolated from human ulcer in Ohio 1941 by Dr. Lee Foshay [43].

## Discussion

In this study, we found that different propagation conditions for the two *F. tularensis* strains FSC198 and FSC043 were supported by genomic data. While propagation in natural conditions has been assumed for the strain FSC198, the strain FSC043 has been extensively passaged in vitro in laboratories. Our results confirm previous findings that FSC198 differs from SCHU S4 at three VNTR loci and by eight intragenic and non-synonymous SNP mutations. In contrast, FSC043 was identical with SCHU S4 at all 25 VNTR loci, and no SNP mutations were found. Instead, all mutations in FSC043 were found to be intragenic deletion events; three micro deletions and the previously identified large deletion RD18 [11].

All four mutations found in strain FSC043 have caused disruption of gene functions: all of the disrupted genes in the strain FSC043 have been linked to virulence or have orthologs in *F. novicida* that have been linked to virulence. One of the two genes (FTT0918) which span the large deletion region RD18, is involved in iron uptake [12] and has been shown to be essential for virulence in the parental SCHU S4 strain [13] as well as in the attenuation of the Live Vaccine Strain [14]. Similar repeat-mediated deletions as in the RD18 locus (and another locus denoted RD19) have been identified, and seem to be characteristic for several laboratory propagated *F. tularensis* strains [11]. In agreement, it has frequently been observed that the genomes of laboratory strains eventually become degraded after passage on artificial media [15].

The Ftind52 and Ftind53 mutations represent identical deletion events but in different copies of the *pdpC* gene of the duplicated *Francisella* Pathogenicity Island, a locus important for *F. tularensis* virulence [16]. While these mutations could have occurred independently, it is likely that the mutation at one *pdpC* locus could have been transferred to the other *pdpC* locus by gene conversion (nonreciprocal recombination). High sequence homogeneity of insertion sequence elements within *F. tularensis* strains but divergence between subspecies suggests a strong effect of this process in *F. tularensis*. The *pdpC* gene is required for infection of *F. tularensis* in mammalian cells [17] but not for *F. novicida* infection of mosquito cells [18]. The mutation Ftind51 affects a putative metal ion transporter protein. A transposon mutant of the corresponding gene in *F. novicida* was negatively selected in a mouse model of infection [19]. The Ftind51 mutation may therefore also be linked to virulence in the strain FSC043. In the strain FSC198, three(*sthA*, *ybhO* and *metA*) of the seven genes affected by mutation have been linked to virulence [20]–[22]. Since approximately 30% of the core genome (1162 genes) in *F. tularensis* [23], have been experimentally identified as virulence genes to date [19]–[22], [24]–[27], it is possible that the seemingly preferential disruption of virulence genes in the FSC043 and the mutation of virulence genes in the FSC198 may be due to chance.

Although certain mutations (e.g. rearrangements, large tandem repeat-polymorphisms) are not reliably detected by the sequencing methodology used, it is not unlikely that the few deletions detected completely may explain the attenuation of virulence in the strain FSC043. This hypothesis, however, needs further examination by specific phenotypic characterization of the strains studied and by experimental gene deletion and/or complementation [28].

Two evolutionary scenarios may have resulted in the gene disruptions detected in the strain FSC043. The disrupted genes may represent neutral events (i.e. genetic drift), caused by genetic bottlenecks that reduced the impact of selection, or because the mutated genes became superfluous when the bacterium was cultured on artificial media. It is also possible that the disruptions have been beneficial and therefore become positively selected. In a recent study of experimental populations of *Escherichia coli* [29], where the impact of genetic drift was reduced by the use of large inoculates, the results indicated positive selection as the predominant cause of the fixation of mutations. Since the strain FSC043 is likely to have experienced reoccurring and severe genetic bottlenecks by the transfer of single colonies between agar plates, however, the fixation of the disruptive mutations could also be due to genetic drift in this strain. Regardless of whether mutations in the FSC043 are neutral (fixed by genetic drift) or non-neutral (fixed by positive selection), it is interesting that the frequency of fixed disruptive mutations (INDELs) occurred at a frequency that greatly exceeded all other mutations (SNPs, VNTRs) in the strain FSC043. This pattern contrasts sharply to that for the naturally propageted strain FSC198, where all mutations were non-synomous SNPs and VNTRs reflecting the adaptation to its propagation environment.

Thus, our data agree with previous indications that the strain FSC198 has been propagated in a natural environment subsequent to its divergence from the progenitor strain SCHU S4 [7]. We find also that these results support the potential utility of analysis of mutational patterns to infer past propagation conditions. The generality and validity of these findings will require further confirmation, but may provide a new type of evidence in microbial forensics.

## Materials and Methods

### Strains

The *F. tularensis* subsp. *tularensis* strain FSC043 was obtained from the *Francisella* Strain Collection (FSC) at the Swedish Defense Research Agency, Umeå. FSC043 was deposited to FSC by the Rocky Mountain Laboratories, Hamilton, MT, US, in 1992. The strain FSC043 represents a standard laboratory strain and it has as such been cultured extensively over the past six decades. It is uncertain precisely when the attenuating genetic mutations occurred. An overview of strains and genome sequences used is presented in Table 2. Major relationships within the species *Francisella* are depicted in Figure 1.

### Genome sequencing of FSC043

FSC043 was re-cultured, suspended in phosphate buffered saline and heat-killed. DNA was prepared by a chaotropic salt method [30]. Sequencing was performed by a commercial service provider (Geneservices, Cambridge, UK) using an Illumina GAII instrument with 36 bp single-end reads. Images acquired from the Illumina sequencer were processed through the Illumina pipeline to obtain sequence and quality scores for each base. Sequence reads have been deposited in the NCBI Short Read Archive [31] as SRA009329.1.

### Genome comparisons

Genome assembly was performed by two alternative, complementary approaches. The first method was based on alignment and assembly using reference genomes from two strains within the subspecies *tularensis* type A1, FSC198 [7] and SCHU S4 [9]. Firstly, sequences from FSC043 were compared against the reference genomes using VAAL [32]. Additional analysis was performed in MOSAIK [33] allowing for non-unique hits in assembly, followed by identification of SNPs and small INDELs using Gigabayes [33]. Allowing non-unique mapping of reads allows identification of potential mutations within duplicated regions. Results from both VAAL and MOSAIK were inspected and confirmed in Consed [34].

Secondly, *de novo* assembly of short reads was performed using Velvet [35] using settings producing the highest N50 value. Constructed contigs were mapped to the same reference genomes using Exonorate [36] and nucmer in the package MUMmer [37]. Identified mutations among the three analyzed type A1 strains were further compared to the type A2 strain WY96-3418 [38] and the type B strain LVS.

Sequence differences around VNTRs and RD18 in *Francisella* genomes (Table 1) were analyzed *in silico* using previously published primers [5]. To confirm VNTRs in FSC043, MLVA was performed using a CEQ 8800 instrument (Beckman Coulters, Fullerton, CA), as previously described [5].

### Verification of mutations within duplicated region

The *F. tularensis* subsp. *tularensis* strains SCHU S4 and FSC043 were grown on modified GC-agar base at 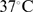 5% [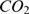], suspended in water and used as PCR (Polymerase Chain Reaction) templates together with Expand Long Range polymerase (Roche Applied Science, Mannheim, Germany). Firstly, the regions FTT1353 to FTT1360 and FTT1709 to FTT1715 of the FPI were amplified using the internal FPI primer pairs FPI-1 and FPI-2, (Table 3), in order to differentiate the two copies of the FPI. Each region comprised approximately 17 kbp. The resulting PCR products were purified from agarose using the High Pure PCR Purification Kit (Roche Applied Science, Mannheim, Germany) according to manufacturer's protocol. A second PCR was performed on each of the two purified PCR products, where the 5.5 kb regions surrounding *pdpC1* and *pdpC2*, respectively, was PCR amplified as eight sequential fragments to facilitate sequencing using primer pairs pdpC-1 to pdpC-8 (Table 3). The average overlap between fragments was 118 nucleotides. Each fragment was cloned into the pCR4-TOPO TA cloning vector (Invitrogen AB, Stockholm, Sweden) and plasmids corresponding to four different clones from each of the eight combinations were purified using the QIAPrep Spin Miniprep Kit (Qiagen, Hilden, Germany) and all 32 clones were sequenced (Eurofins MWG operon, Ebersberg, Germany). A one base pair deletion was observed in both copies of *pdpC* in FSC043. To verify this, the region was amplified in both SCHU S4 and FSC043 using primer pair pdpC-9 (this does not allow a separation of the two FPI copies), and subsequent sequencing of the 691 bp PCR product was performed, confirming the mutation. No other differences were observed among the 5.5 kb sequenced region.

**Table 3:** Primers used to amplify regions surrounding *pdpC1* and *pdpC2* to confirm the Ftind52 and Ftind53 mutations.

Region name	Forward primer (5′ 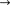 3′)	Reverse primer (5′ 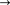 3′)
FPI-1	CCAGAATGACCCCGTAGAAA	CTGCCTCAAAAAGCTCACCT
FPI-2	CCAGAATGACCCCGTAGAAA	TGCTGTAGCTCATGGTGAGG
pdpC-1	TACTAAAGCACTGCACAAATTCAC	GCATCGCTATTTTTGAGGGA
pdpC-2	GCATGTATGAGGAGATTAAGAGC	TGTCTTACGACTAGCGCGTCTA
pdpC-3	CGAGGGGTTACTTGAAAATCT	GAAGCCAGGAAGATAGCATACT
pdpC-4	TCCTGGCTTCTTGAGCTCTGTAA	TGATCGACACTATGTGCCATG
pdpC-5	ACTCGGGATGGCAACTACAA	TCTGAATTAGGTGTTGCGAAACT
pdpC-6	GATTTCAACTTATACTCACCAGA	TCTATTACTGGTTTTGAGGCTC
pdpC-7	TGTGGATTAGTAGCGAAATTGTA	GTTCGAATGTACTAGCTATTATTGT
pdpC-8	GCCTTTCACACTAAGCTTATAAC	TGATAGCATTGAATTTGATTTCC
pdpC-9	TCCCTCAAAAATAGCGATGC	TGTCTTACGACTAGCGCGTCTA
